# Progress in Charge Transfer in 2D Metal Halide Perovskite Heterojunctions: A Review

**DOI:** 10.3390/ma18245690

**Published:** 2025-12-18

**Authors:** Chenjing Quan, Jiahe Yan, Xiaofeng Liu, Qing Lin, Beibei Xu, Jianrong Qiu

**Affiliations:** 1 State Key Laboratory of Extreme Photonics and Instrumentation, College of Optical Science and Engineering, Zhejiang University, Hangzhou 310027, China; quanchenjingnlo@163.com; 2 School of Materials Science and Engineering, Zhejiang University, Hangzhou 310058, China; 12326060@zju.edu.cn; 3 Jiangsu Engineering Research Center of Key Technology for Intelligent Manufacturing Equipment, School of Mechanical and Electrical Engineering, Suqian University, Suqian 223800, China; linqing@squ.edu.cn

**Keywords:** metal halide perovskite (MHP), heterojunction, carrier dynamics, charge separation and transfer, ultrafast spectroscopy, optoelectronic devices

## Abstract

**Highlights:**

**What are the main findings?**

- This review summarizes charge transfer mechanisms and interfacial coupling effects in two-dimensional (2D) metal halide perovskite (MHP)-based heterojunctions, with a focus on their roles in governing device performance.
- Ultrafast carrier dynamics and band-engineering strategies are systematically discussed, highlighting effective approaches to enhance charge separation and suppress nonradiative recombination.

**What are the implications of the main findings?**

- Interface engineering and molecular passivation are identified as key routes to improving the stability and operational performance of 2D MHP-based devices.
- The presented insights provide guidance for the rational design of flexible, high-efficiency optoelectronic and quantum photonic devices.

**Abstract:**

Metal halide perovskite (MHP)-based heterojunctions have become a forefront area in the research of optoelectronic functional materials due to their unique layered crystal structure, tunable band gaps, and exceptional optoelectronic properties. Recent studies have demonstrated that interface charge transfer is a crucial factor in determining the optoelectronic performance of the heterojunction devices. By constructing heterojunctions between MHPs and two-dimensional (2D) materials such as graphene, MoS_2_, and WS_2_, efficient electron–hole separation and transport can be achieved, significantly extending carrier lifetimes and suppressing non-radiative recombination. This results in enhanced response speed and energy conversion efficiency in photodetectors, photovoltaic devices, and light-emitting devices (LEDs). In these heterojunctions, the thickness of the MHP layer, interface defect density, and band alignment significantly influence carrier dynamics. Furthermore, techniques such as interface engineering, molecular passivation, and band engineering can effectively optimize charge separation efficiency and improve device stability. The integration of multilayer heterojunctions and flexible designs also presents new opportunities for expanding the functionality of high-performance optoelectronic devices. In this review, we systematically summarize the charge transfer mechanisms in MHP-based heterojunctions and highlight recent advances in their optoelectronic applications. Particular emphasis is placed on the influence of interfacial coupling on carrier generation, transport, and recombination dynamics. Furthermore, the ultrafast dynamic behaviors and band-engineering strategies in representative heterojunctions are elaborated, together with key factors and approaches for enhancing charge transfer efficiency. Finally, the potential of MHP heterojunctions for high-performance optoelectronic devices and emerging photonic systems is discussed. This review aims to provide a comprehensive theoretical and experimental reference for future research and to offer new insights into the rational design and application of flexible optoelectronics, photovoltaics, light-emitting devices, and quantum photonic technologies.

## 1. Introduction

Since its discovery by Gustav Rose in the Ural Mountains in 1839, CaTiO_3_ has been named “Perovskite” in honor of Russian geologist Lev Perovski, marking the birth of the perovskite material [1]. It was not until 1893 that CsPbX_3_ was first discovered [2]. In recent years, metal halide perovskites (MHPs) have garnered significant attention in the field of optoelectronic materials due to their excellent optoelectronic properties, such as tunable bandgap, high optical absorption coefficient, long carrier lifetimes, and high photoluminescence quantum yield (PLQY) [3,4,5]. MHPs have been successfully applied in high-efficiency solar cells [6,7], light-emitting diodes (LEDs) [8,9], photodetectors [10], and lasers [11]. By adjusting their composition and structure, the emission wavelength can be tuned continuously from near-infrared to visible regions [12,13,14,15,16], while their high PLQY makes them ideal gain media for LEDs and micro/nano lasers [7,17,18]. It has been shown that the generation time of electron-hole pairs in MHPs upon light absorption is less than 1 ps, while their recombination lifetime can reach the microsecond scale [19]. This unique combination of ultrafast excitation and long lifetimes gives MHPs a distinctive advantage in optoelectronic devices with high response speeds and energy utilization efficiency [20]. Therefore, understanding the generation, migration, and recombination mechanisms of photogenerated carriers in MHPs is crucial for comprehending their optoelectronic performance and improving device efficiency.

To further improve device performance and deepen the understanding of the excited-state process, researchers have proposed the combination of MHPs with high-mobility two-dimensional (2D) materials to construct van der Waals (vdWs) heterojunctions for efficient charge separation and transport. Materials such as graphene [21], hexagonal boron nitride (h-BN) [22], and transition metal dichalcogenides (TMDs) [23,24], with their weak interlayer interactions, can be mechanically exfoliated to obtain high-quality 2D layers. Because the interactions between vdWs heterojunctions are not constrained by lattice mis-matching, the band structures and interface electronic states of vdWs are highly tunable. The optoelectronic properties of these heterojunctions primarily depend on the interface charge transfer and energy transfer behaviors, which are influenced by factors such as the type of band alignment, relative dielectric constant, stacking arrangement, crystal orientation, as well as external electric, optical, and strain fields [25,26]. The integration of MHPs with 2D materials not only leverages the properties of each material but also enhances performance through interface coupling, leading to significant improvements in both optoelectronic conversion efficiency and device stability.

Similarly to other inorganic direct-bandgap semiconductors, MHP exhibits tunable electronic structural characteristics and can be used to fabricate stable heterojunctions with 2D materials via vdW forces. For instance, in MHP/graphene heterojunctions, graphene can act as an electron transport layer (ETL), while the perovskite layer serves as a high-absorption layer, effectively promoting charge separation and improving carrier collection efficiency [27,28,29]. However, graphene’s low absorption cross-section, rapid recombination rate, and lack of optical gain mechanisms still limit its independent application in optoelectronic devices. Therefore, rationally designing the interface coupling between MHPs and 2D materials to precisely control charge transfer rates and carrier dynamics has become a key direction for enhancing optoelectronic device performance. The ultrafast carrier dynamics in MHP heterojunctions typically occur on the femtosecond to picosecond time scale, involving key processes such as exciton dissociation, free carrier transport, and interlayer charge transfer. These non-equilibrium processes directly determine the carrier lifetime, mobility, and quantum efficiency of the materials, thereby affecting the response speed and energy conversion efficiency of the devices [15,16]. Utilizing ultrafast spectroscopic techniques, such as time-resolved transient absorption (TA) spectroscopy [30,31,32], time-resolved terahertz (THz) spectroscopy (TRTS) [33,34,35,36], optical pump-THz probe technology (OPTP) [37] and time-resolved photoluminescence spectroscopy (TRPL) [38,39], allows for in-depth analysis of these dynamic processes, providing experimental insights into understanding the interface physics and enabling efficient control strategies.

In this review, we concentrate our discussion on the charge transfer mechanisms in MHP heterojunctions and their applications in optoelectronic devices. We present a comprehensive review on the impact of heterojunction interface coupling on carrier generation, migration, and recombination processes, the ultrafast dynamics characteristics and band engineering strategies in typical systems, the key factors and methods for controlling charge transfer efficiency, and the prospects of perovskite heterojunctions in high-efficiency optoelectronic devices and novel photonic systems.

## 2. Architecture of Metal Halide Perovskite-Based Heterojunctions

**Figure 1:**
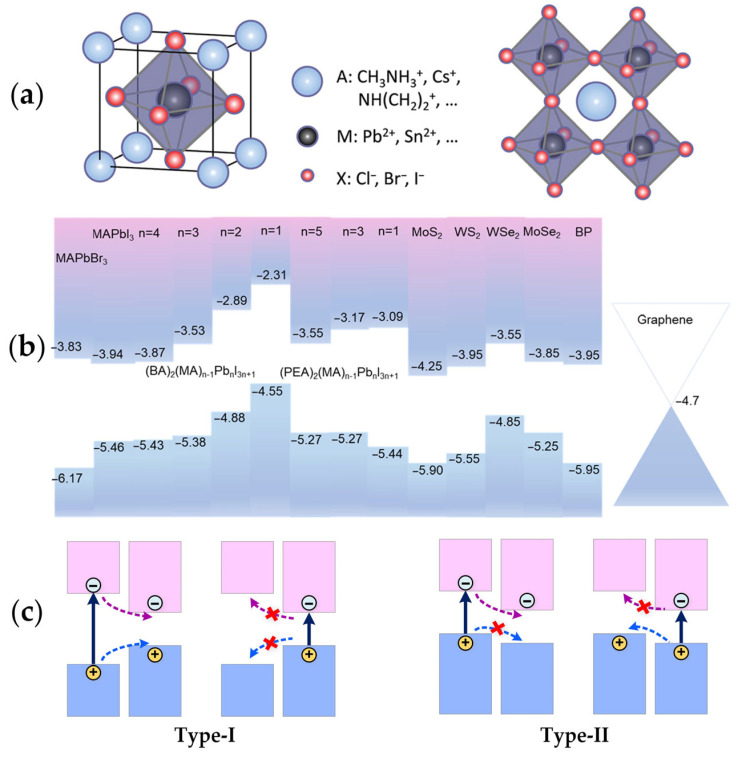
Schematic illustration of the atomic structure of MHPs and the band structures of vdW heterojunctions. (**a**) Crystal cell and crystal structure diagram of MHPs [40]; (**b**) electronic band structures of MHPs with different n-value cations, TMDs, and graphene [43]; (**c**) schematic diagrams of type-I and type-II band alignments and the corresponding charge transfer directions.

MHPs generally adopt the formula of AMX_3_, as shown in Figure 1a. In this structure, the A-site is typically occupied by organic or inorganic cations (e.g., Cs^+^, CH_3_NH_3_^+^ (MA^+^), (C_2_H_5_)_4_N^+^ (TEA^+^), C_6_H_5_C_2_H_4_NH_3_^+^ (PEA^+^), C_4_H_9_NH_3_^+^ (BA^+^), CH(NH_2_)_2_^+^ (FA^+^), NH(CH_2_)_2_^+^, and etc.). The M-site is occupied by divalent metal cations such as Pb^2+^ and Sn^2+^, while the X-site is taken by halide anions (Cl^−^, Br^−^, I^−^) or mixed halide ions. Due to the different hybridization energies of halogen atomic orbitals, the bandgap of perovskite materials can be tuned by altering the elemental composition [40,41].

By adjusting the composition of MHP and the number of layers in 2D heterojunctions, the charge transfer at the heterojunction interface and other processes can be further controlled. This enables the induction of novel optoelectronic properties through interlayer coupling effects, thereby extending the functionality of optoelectronic devices [25]. As shown in Figure 1b, the electronic band alignments of MHP materials with different n-values of cations, TMDs, and graphene can form two types of band alignments, type-I and type-II, due to differences in the work functions between 2D semiconductor materials. In Figure 1c, when both the conduction band minimum (CBM) and valence band maximum (VBM) exist in a material with a narrower bandgap, it can form a type-I heterojunction when combined with a material with a wider bandgap [42]. After light excitation in a type-I heterojunction, electrons and holes are generated in the wide-bandgap material and injected into the narrow-bandgap semiconductor, as indicated by the arrows. However, due to the lower energy, the carriers excited in the narrow-bandgap material cannot undergo interlayer transfer. The quantum confinement of these electrons and holes in the same region enhances radiative recombination, making it beneficial for light-emitting devices.

In contrast, the CBM and VBM are located in different constituent materials on opposite sides of the type-II heterojunction. Upon photoexcitation in the wide-bandgap component, electrons are transferred across the interface, whereas excitation within the narrow-bandgap component induces hole transfer. This staggered band alignment results in spatial separation of electrons and holes into distinct layers within the heterojunction, thereby suppressing recombination and extending carrier lifetimes. Such characteristics render type-II heterojunctions highly promising for applications in photovoltaic and photodetector devices [36,42,44].

## 3. Ultrafast Optical Processes in Metal Halide Perovskite Heterojunctions

### 3.1. Ultrafast Spectroscopic Techniques

Compared with traditional semiconductors, where carrier cooling occurs extremely rapidly, MHPs exhibit significantly prolonged hot-carrier lifetimes, offering distinct advantages for controlling hot-carrier relaxation. The mechanisms behind the slowed cooling of hot carriers in perovskites remain complex, involving processes such as the hot phonon bottleneck [45,46] and Auger heating [47,48]. Prolonging carrier lifetime is one of the key factors for improving the conversion efficiency of MHP optoelectronic devices, which is typically evaluated through parameters such as carrier diffusion length [49,50,51,52].

**Figure 2:**
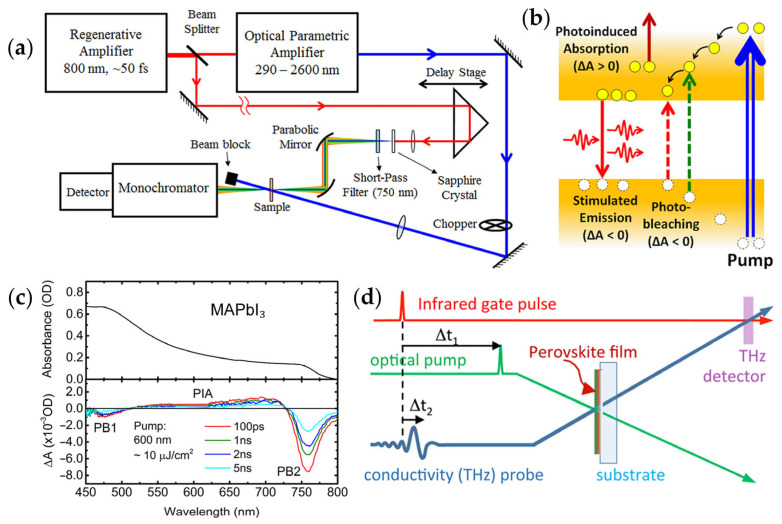
Schematic overview of carrier relaxation processes and corresponding ultrafast spectroscopic characterization techniques. (**a**) Schematic diagram of a non-degenerate time-resolved TA setup and (**b**) illustration of PB, PIA and SE processes in TA measurements [20]. (**c**) Linear absorption spectrum (top) and TA spectra under 600 nm excitation (bottom) of MAPbI_3_ [53]. (**d**) Schematic representation of pulsed laser beam layout for the OPTP technique [37].

TA spectroscopy is one of the core techniques for studying the evolution of nonequilibrium states following optical excitation. As illustrated in Figure 2a, a femtosecond laser generates a pump pulse through an optical parametric amplifier (OPA) to excite the sample. The probe light is obtained from a white light continuum produced by a small portion of the pump light focused onto a sapphire crystal. By varying the delay between the pump and probe pulses, information about the evolution of the absorption spectrum with time can be obtained. Figure 2b illustrates the three main components of the TA signal [20]: photobleaching (PB), stimulated emission (SE), and photoinduced absorption (PIA). In the experiment, the detector alternately measures the transmitted signal with the pump light on (I(λ)_pump-on_) and off (I(λ_)pump-off_), and the differential absorption spectrum is defined as (ΔA(λ) = −log(I(λ)_pump-on_/I(λ)_pump-off_)). PB (ΔA < 0) arises from the depletion of ground-state electrons after excitation, leading to reduced absorption; SE (ΔA < 0) corresponds to stimulated emission caused by transitions from the excited state to the ground state; while PIA (ΔA > 0) reflects new absorption channels resulting from the further transition of excited-state electrons to higher energy levels. As shown in Figure 2c, the linear absorption (top) and TA spectra of MAPbI_3_ was measured using a 600 nm pump with ~10 μJ/cm^2^ fluence (bottom). Two prominent PB features are observed at ~480 nm (PB1) and ~760 nm (PB2), accompanied by a broad positive ΔA band between 550 and 650 nm, attributed to photoinduced refractive index changes. PB is associated with the band-edge absorption or excitonic transitions [37]. Thus, TA spectroscopy enables comprehensive probing of photoinduced carrier and exciton dynamics at both spectral and temporal dimensions, offering vital insights into ultrafast optical response mechanisms.

TRTS provides a powerful tool for studying photogenerated carrier transport. Free carriers significantly alter the low-frequency dielectric response of materials, and THz spectroscopy is highly sensitive to the dynamic processes of carrier generation, migration, and recombination. As shown in Figure 2d, OPTP combines femtosecond time resolution with the advantages of non-contact measurement. The time resolution is limited by the sub-picosecond pulse durations, and the full amplitude waveform of the THz pulse can be recorded, from which the time-dependent complex photoconductivity spectrum in the THz region can be extracted, enabling detailed analysis of conduction mechanisms. By monitoring changes in the THz transmission signal (ΔT/T) after optical excitation, this measurement directly provides transient photoconductive properties of the material. OPTP technique not only allows the extraction of key parameters such as carrier mobility and lifetime but also reveals interface charge transfer and recombination dynamics [36,37]. Consequently, MHP heterojunctions present an ideal platform for elucidating the photophysical mechanisms that render these materials highly efficient as light absorbers and charge conductors.

In addition, TRPL is another essential technique for investigating carrier dynamics. Using a pulsed femtosecond or picosecond laser, TRPL monitors the temporal decay of photoluminescence intensity to reveal carrier relaxation and recombination pathways. The emitted photons are spectrally dispersed by a monochromator and detected using time-correlated single-photon counting or a streak camera, enabling sub-picosecond to nanosecond temporal resolution. Typically, TRPL decay curves consist of fast and slow components corresponding to nonradiative (trap-assisted) and radiative (free carrier or excitonic) recombination, respectively. By analyzing TRPL dynamics under different excitation powers, temperatures, or device structures, one can quantitatively evaluate carrier lifetimes, interfacial charge transfer rates, and defect-related recombination pathways. Therefore, TRPL provides crucial information for understanding carrier dynamics and optimizing interfacial properties in MHP heterojunction optoelectronic materials.

As mentioned, the carrier relaxation and interface transfer processes in MHPs play a crucial role in determining the optoelectronic performance of devices. However, research on carrier dynamics in MHP heterojunctions with other low-dimensional semiconductors is still relatively limited. A deeper understanding of the interfacial charge transfer and energy evolution mechanisms is essential for optimizing optoelectronic conversion efficiency and designing high-performance heterojunction devices.

### 3.2. Metal Halide Perovskite/Graphene Heterojunctions

Graphene possesses exceptional electrical conductivity, ultrahigh carrier mobility, atomic-level thickness for forming vdW contacts, and excellent transparency, making it an outstanding candidate for transparent electrodes and interfacial engineering in metal halide perovskite solar cells (PSCs) [54,55]. Numerous studies have demonstrated that incorporating graphene or its derivatives into PSCs can significantly improve device performance and stability [56].

In MHP/graphene heterojunctions, rapid carrier extraction, suppressed interfacial recombination, improved energy-level alignment, reduced series resistance, and enhanced environmental stability can theoretically be achieved [56]. Graphene serves not only as a transparent conductive electrode or carrier transport layer but also as an interfacial modification layer, such as inserted graphene, graphene oxide, or reduced graphene oxide, to passivate interfacial defects and improve device stability [28,29,57]. When graphene forms a direct vdW contact with the perovskite layer, a true heterojunction is realized, where interfacial charge transfer and band alignment directly determine device performance.

For instance, Wang et al. [58] fabricated graphene/perovskite heterojunction phototransistors using a rapid-crystallization deposition technique. The resulting devices delivered an exceptionally high photoresponse (~6.0 × 10^5^ A W^−1^) together with a photoconductive gain on the order of 10^9^. Moreover, the pronounced PL quenching and reduced carrier lifetimes observed in these structures provide clear evidence of highly efficient charge transfer across the graphene/perovskite interface. Upon illumination, the perovskite layer absorbs photons to generate electron–hole pairs, which separate under the built-in electric field: holes migrate to the graphene layer, while electrons remain trapped within the perovskite, exhibiting longer lifetimes. This process enables multiple recirculation of positive charges (holes) within the graphene layer, significantly enhancing conductivity and optical gain, thereby achieving high-sensitivity optoelectronic conversion.

Under femtosecond laser excitation, the heterojunction exhibits a pronounced THz signal enhancement within several hundred femtoseconds, followed by rapid decay. This transient optical response reflects the dynamic evolution of positive and negative conductivities over time, resulting from the synergistic effects of Fermi-level shifts in graphene and interfacial charge transfer dynamics [25]. Nevertheless, several challenges remain. The fabrication and transfer of high-quality graphene layers with uniform coverage, control of interfacial defects, and chemical compatibility with perovskite materials require further optimization [59]. Additionally, thermal expansion mismatch and interfacial strain may influence perovskite film morphology and the stability of large-area devices [25]. Addressing these issues will be critical for the scalable fabrication of high-performance perovskite/graphene heterojunction devices.

### 3.3. Metal Halide Perovskite/TMDs Heterojunctions

The combination of MHPs with 2D TMDs enables the formation of heterojunctions, providing an ideal platform to investigate photogenerated carrier dynamics due to their excellent optoelectronic properties and tunable band structures. Early investigations established the fundamental charge-transfer behaviors in such systems. For instance, Fang et al. [27] constructed vertical heterojunctions of CsPbBr_3_ nanowires with monolayer MoS_2_ and WSe_2_, and investigated their ultrafast carrier dynamics using TA and TRPL spectroscopy. The results show that the CsPbBr_3_/MoS_2_ heterojunction forms a type-II band alignment, enabling bidirectional carrier injection from perovskite to MoS_2_ with an overall transfer efficiency of ~71%. In CsPbBr_3_/WSe_2_ heterojunction, also type-II, holes transfer from perovskite to WSe_2_, while electrons return to the perovskite within ~7 ps, realizing spatially separated carrier transport with an efficiency of ~70%. These results confirm the strong interfacial coupling and efficient charge separation achievable in MHP/TMD systems.

**Figure 3:**
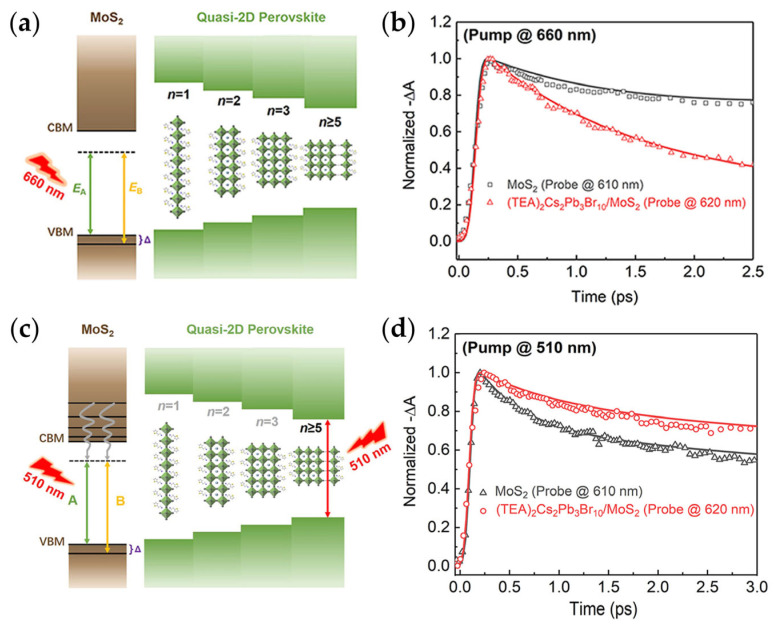
Schematic illustration of charge transfer in 2D MHP/TMDs Heterojunctions. Energy band alignments and corresponding TA dynamics of (TEA)_2_Cs_2_Pb_3_Br_10_/MoS_2_ heterojunction under (**a**,**b**) 660 nm and (**c**,**d**) 510 nm excitation [60].

Building upon these insights, Qin et al. [60] further elucidated the ultrafast interfacial charge dynamics in the (TEA)_2_Cs_2_Pb_3_Br_10_/MoS_2_ heterojunction using TA spectroscopy. When selectively exciting monolayer MoS_2_ at 660 nm (targeting the A-exciton), the B-exciton signal (610 nm) in MoS_2_ decayed more rapidly than that of the heterojunction (620 nm) (Figure 3a,b). The heterojunction exhibited a fast decay component (τ_1_ = 0.38 ps) and a slow component (τ_2_ = 12.5 ps), both shorter than those of isolated MoS_2_ (τ_1_ = 0.71 ps, τ_2_ = 20.2 ps). A rise time of 28.3 ps was observed, indicating hole transfer from MoS_2_ to (TEA)_2_Cs_2_Pb_3_Br_10_ within this timescale. Under 510 nm excitation of both layers, the heterojunction showed an extended decay and a formation time of 0.12 ps, demonstrating electron transfer from (TEA)_2_Cs_2_Pb_3_Br_10_ to MoS_2_ (Figure 3c,d).

To further elucidate the structure and property correlation, Huang et al. [61] further investigated the thickness-dependent charge transfer in (BA)_2_PbI_4_/WS_2_ heterojunction using TRPL spectroscopy. A heterojunction with thicker (BA)_2_PbI_4_ layers exhibited weaker carrier lifetime modulation due to longer carrier transport paths and enhanced recombination before reaching the interface. Conversely, thinner (BA)_2_PbI_4_ layers facilitated more efficient carrier transfer to monolayer WS_2_, leading to a greater lifetime reduction and strong PL quenching.

Overall, these studies collectively demonstrate that the efficiency and directionality of interlayer charge transfer in MHP/TMDs heterojunctions are highly tunable through band alignment engineering and layer thickness modulation, providing valuable design guidelines for high-performance optoelectronic and photovoltaic devices.

### 3.4. Metal Halide Perovskite/Metal Oxide Heterojunctions

Building on the insights gained from MHP/TMDs heterojunctions and their regulation of carrier dynamics, further exploration of heterojunctions formed between MHPs and metal oxides (e.g., TiO_2_, SnO_2_, NiO_x_) can help optimize interfacial charge injection, extraction, and transport, thereby offering new approaches for designing high-performance optoelectronic devices.

In 2016, Deng et al. [62] used TAs and TRTS to observe an ultrafast thermalization process of hot carriers in MAPbBr_3_ films upon optical excitation. They found that free electron-hole pair recombination and Auger recombination were the primary relaxation processes for the carriers. At the MAPbBr_3_/TiO_2_ interface, the electron transfer time was measured to be 0.68 ns, during which TiO_2_ effectively collected the excited electrons. This efficiency is attributed to the high-quality MHP films, which offer enhanced stability, increased light absorption, and a higher photogenerated carrier density. The choice of an appropriate ETL can significantly extend carrier lifetimes, providing a theoretical foundation for understanding carrier relaxation and transport dynamics in MHPs and for optimizing MHP-based devices.

**Figure 4:**
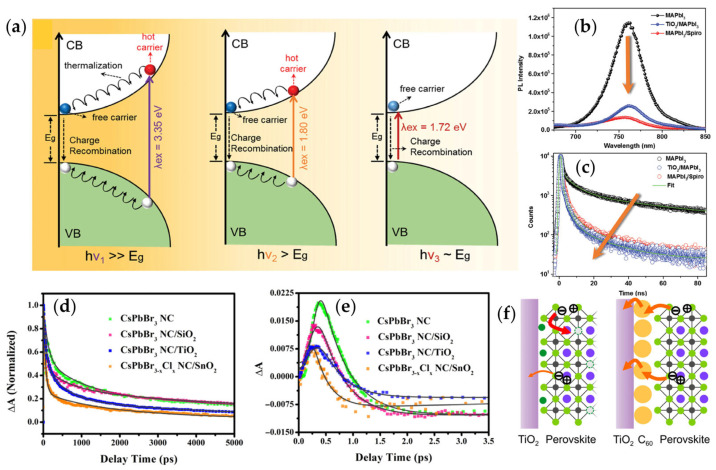
Hot-hole extraction in MHP-based heterojunctions. (**a**) Schematic of electron relaxation processes in MAPbI_3_ under three pump excitations: excess energy, near the band edge, and at the band edge; (**b**) steady-state PL spectra and (**c**) TRPL spectra of pristine MAPbI_3_ (black), TiO_2_/MAPbI_3_ (blue), and MAPbI_3_/spiro-OMeTAD (red) [63]. TA spectra of the MAPbI_3_/TiO_2_ films [66]. TA dynamics probed at the position of (**d**) PB and (**e**) PIA features under 400 nm pump pulse of CsPbBr_3_ nanocrystal (NC), CsPbBr_3_ NC/SiO_2_, CsPbBr_3_ NC/TiO_2_, and CsPbBr_3−x_Cl_x_ NC/SnO_2_ [67]. (**f**) Schematic illustration of the carrier collection and hot electron extraction across MAPbI_3_, C_60_, and TiO_2_ Interfaces [68].

Using TA spectroscopy and density functional theory (DFT) modeling, Dursun et al. [63] investigate hot carrier dynamics and extraction at the MAPbI_3_ perovskite interfaces with TiO_2_ and spiro-OMeTAD. Unlike TiO_2_, spiro-OMeTAD prevents spectral broadening of hot carriers, indicating efficient hole extraction to the hole transport layer (HTL). The DFT modeling shows this extraction is driven by the strong overlap between spiro-OMeTAD’s valence band charge and the delocalized electronic states on MAPbI_3_’s surface. Figure 4a illustrates the impact of excitation energy on carrier dynamics in MAPbI_3_. Excitation with excess energy excites charge carriers to hot states, which recombine and thermalize on timescales >5 ns. Band-edge excitation reveals free carrier recombination, providing the true carrier lifetime (~ns). Thermalization occurs primarily via LO phonon emission, with recent studies highlighting spectral broadening due to Fröhlich interaction-induced phonon scattering [64] and electron-phonon coupling during electron transport [65].

The steady-state PL spectra in Figure 4b show PL quenching after introducing either an ETL or HTL, highlighting highly efficient extraction of thermalized carriers from MAPbI_3_ [63]. Consistently, TRPL measurements (Figure 4c) reveal a pronounced reduction in the radiative lifetime of MAPbI_3_ in the presence of ETL (0.68 ± 0.04 ns) and HTL (0.76 ± 0.04 ns), further confirming the accelerated depopulation of photoexcited carriers. These observations collectively underscore the fundamental importance of carrier extraction efficiency in dictating the performance of perovskite photovoltaic devices.

Building on these insights, recent studies have increasingly focused on tailoring interfacial structures to regulate hot-carrier cooling and charge-transfer dynamics in PNCs and thin films. Liao et al. [67] conducted a comprehensive investigation into the hot-carrier relaxation and charge-transfer behavior of CsPbBr_3_ NCs coated with different MO_2_ layers (M = Si, Ti, Sn). As shown by the kinetic traces in Figure 4d,e, the hot-carrier cooling time decreased from 482 fs (bare NCs) to 436 fs (SiO_2_), 389 fs (TiO_2_), and ultimately 277 fs when CsPbBr_3−x_Cl_x_ NCs were interfaced with SnO_2_. Recovery-dynamics analysis further yielded electron-transfer rate constants of 5.4 × 10^7^ s^−1^ for CsPbBr_3_/TiO_2_ and 1.14 × 10^8^ s^−1^ for CsPbBr_3−x_Cl_x_/SnO_2_, confirming that SnO_2_ provides a substantially more efficient electron-accepting interface. In addition, CsPbBr_3_/SnO_2_ heterostructures exhibited improved UV/moisture stability and enhanced photocurrent output, demonstrating the dual advantages of surface-coating engineering in electronic-structure modulation and environmental robustness.

Interfacial engineering has also been shown to regulate charge-transfer kinetics across broader temporal regimes. As illustrated in Figure 4f, Jimenez-Lopez et al. [68] introduced a 30 nm C_60_ interlayer between TiO_2_ and MAPbI_3_, which effectively suppressed I–V hysteresis while facilitating carrier injection and recombination. Ultrafast-to-steady-state analyses revealed that C_60_ accelerates hot-carrier extraction immediately after photoexcitation and tunes interfacial charge-transfer lifetimes. Parallel work by Wang et al. [54] adopted a distinct approach by inserting a thin Au layer, enabling ballistic hot-electron transfer from MAPbI_3_ to TiO_2_ and yielding a short-circuit current density of 5.422 mA cm^−2^ under standard illumination.

Collectively, these studies demonstrate the pivotal role of interface engineering in tailoring hot-carrier relaxation, enhancing charge-transfer efficiency, and ultimately improving the performance of perovskite-based optoelectronic devices.

## 4. Applications of Metal Halide Perovskite-Based Heterojunctions in Optoelectronic Devices

### 4.1. Solar Cells

**Figure 5:**
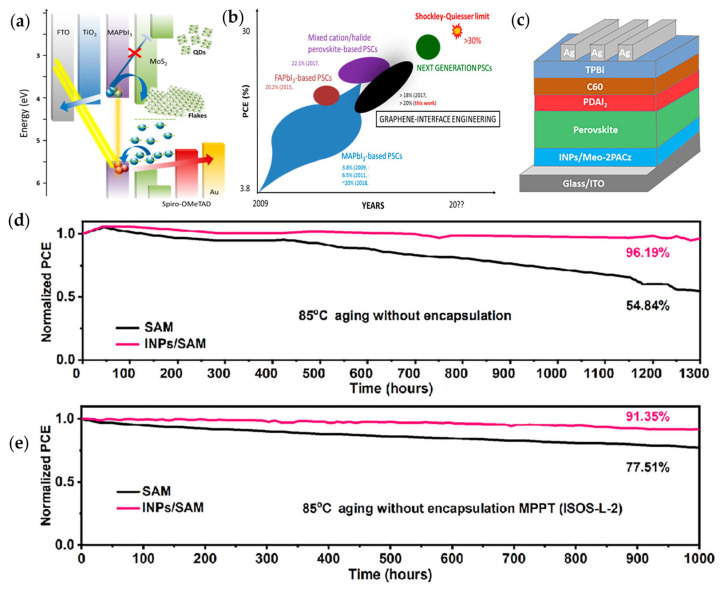
Representative PSCs and their performance characteristics. (**a**) Energy band structure schematic of the MAPbI_3_-based solar cell; (**b**) Performance comparison of perovskite solar cells enhanced by interfacial engineering and chemical synergistic strategies [57]. Spiro-MeOTAD-based PSC device and its encapsulated operational stability, including thermal stability at (**c**) Device structure of inverted PSCs. (**d**) Thermal stability of PSCs at 85 °C in N_2_ environment. (**e**) Operational stability of PSCs under maximum power point tracking with continuous illumination at 85 °C [79].

In recent years, research on metal halide PSCs has focused on materials, device structures, and fabrication techniques to enhance performance and understanding. Interface engineering plays a crucial role in optimizing PSC performance. Graphene and related 2D materials, due to their tunable interfacial properties, have emerged as ideal candidates for HTLs or active buffer layers. As shown in Figure 5a, MoS_2_ quantum dots (QDs) combine hole extraction and electron-blocking functions, with quantum confinement widening the bandgap from 1.4 eV to over 3.2 eV and raising the conduction band minimum to −2.2 eV, above the perovskite level. Hybrid vdW structures of MoS_2_ QDs and MPTS-functionalized reduced graphene oxide form uniform, pinhole-free interlayers, enabling MAPbI_3_ PSCs to achieve a maximum power conversion efficiency (PCE) of 20.12% (average 18.8%) while suppressing interfacial recombination [57]. Similar approaches using graphene-flake- and QD-doped ETLs, including mesoscopic TiO_2_ and solution-processed SnO_2_, further demonstrate the versatility of graphene interface engineering for high-performance PSCs.

As illustrated in Figure 5b, MAPbI_3_ itself has long served as a benchmark light-harvesting material, from the earliest stages of PSC development. Elemental composition engineering led to mixed-cation and mixed-halide perovskites, pushing certified PCEs above 22% (22.1% and 22.7%) [69,70]. While archetypal MAPbI_3_ reached a certified maximum efficiency of 19.3% [71], (with non-certified values exceeding 20%) [72,73], it remains a reference system for optimizing and validating PSC architectures due to its historical impact on the field (starting from a PCE of 3.8% in 2009) [74]. Recently, according to the latest report, Chen’s group achieved a power conversion efficiency of 26.31% by introducing organic cation halide salts to form buried 2D perovskite layers [75]. MHP-based PSCs provide an ideal platform to study and design interfaces between functional layers, as carrier transport barriers at these interfaces govern undesirable hysteresis and the stability issues in both mesoscopic and planar structures. As PSCs approach their theoretical efficiency limit (~31% [76], with practical limits of 29.5–30.5% when accounting for intrinsic nonradiative recombination) [77,78]. graphene and other 2D materials are emerging as a paradigm-shifting tool for interface engineering to further boost photovoltaic performance.

Despite remarkable efficiency improvements, MHPs remain vulnerable to degradation when exposed to moisture, elevated temperatures, oxygen, and ultraviolet irradiation. This persistent instability underscores the need for refined structural designs and chemical engineering strategies, particularly for 2D MHPs. In 2024, Ren et al. [6] reported a mild and precisely controlled SnO_2_ deposition route using SnSO_4_ as the precursor, which is fully compatible with acid-sensitive flexible ITO substrates. The resulting compact and uniform SnO_2_ electron-transport layers enabled flexible PSCs to achieve a PCE of 25.09% (certified 24.90%), establishing a new performance benchmark for flexible perovskite solar technologies.

Using a vacuum-assisted solution process, Zhang et al. [59] realized a certified stable PCE of 17.85%, with devices maintaining over 90% of their efficiency under continuous illumination or prolonged exposure to high-humidity and 85 °C conditions.

Parallel advances in ETLs have also contributed significantly to flexible PSC performance. Tang et al. [80] further employed atomic-layer deposition (ALD) to enhance the stability of self-assembled monolayers (SAMs) used as hole-transport layers. By depositing an additional indium oxide layer via ALD, the SAM molecules were more securely anchored to the ITO surface through strong chemisorption involving the trimethoxysilane groups. The resulting inverted PSCs demonstrated outstanding thermal operation stability, retaining 98% of their initial efficiency under maximum power point tracking (MPPT) at 85 °C for 1200 h. Although SAM-based inverted PSCs have rapidly improved in efficiency, the weak interfacial anchoring of SAM molecules remains a key limitation for long-term stability. To address this, functionalized indium tin oxide nanoparticles (INPs) were incorporated to promote uniform SAM assembly on the electrode surface (Figure 5c). The INPs supply a high density of robustly bonded –OH groups that serve as strong anchoring sites, effectively mitigating SAM detachment during aging and ensuring durable interfacial contact. Consequently, INP-integrated PSCs (Figure 5d,e) achieved an efficiency of 26.44% and exhibited exceptional operational durability, retaining approximately 91% of their initial PCE after continuous MPPT operation at 85 °C [79].

Overall, the synergistic integration of 2D perovskite architectures with advanced electron-transport strategies offers a powerful pathway toward simultaneously enhancing efficiency, operational stability, and mechanical flexibility. These progresses collectively advance the development of high-performance, durable, and flexible perovskite photovoltaic technologies suitable for practical deployment.

### 4.2. Photodetectors

MHPs have demonstrated remarkable potential in photodetection owing to their high solar conversion efficiency, strong optical absorption, and tunable electronic properties. From thin films to single crystals, a series of MHP-based materials has been gradually explored for detecting visible light as well as X-rays and gamma rays. However, bulk films composed of submicron or nanometer-sized perovskite particles introduce a large number of grain boundaries, creating numerous scattering centers for electrons and photons. This leads to issues such as low efficiency and poor responsiveness. Addressing these challenges is crucial for improving the performance of MHP-based photodetectors. The use of high-quality 2D MHPs as the optically active medium in photodetectors has proven to be an effective solution.

Typically, 2D MHP photodetectors operate via two primary mechanisms: the photoconductive effect and the photovoltaic effect. In the photoconductive mode, light absorption generates additional free carriers, thereby reducing the resistance of the material. Remarkable progress has been achieved in 2D MHP/graphene heterojunctions, where the synergistic combination of strong light absorption (MHPs) and high carrier mobility (graphene) leads to ultrahigh responsivity, enhanced external quantum efficiency (EQE), and outstanding photodetection performance [81,82]. The interface of the MHP/graphene heterojunction exhibits efficient charge separation, demonstrating great potential for applications in photodetectors, light-emitting devices, and photovoltaic devices. For example, Seo et al. [83] firstly demonstrated flexible MHP/graphene LEDs in 2017, achieving both high luminescent performance and excellent mechanical flexibility. Subsequently, Lee et al. [84] developed flexible photodetectors that maintained stable response even after 3000 bending cycles (0.5% strain), with an EQE of 5 × 10^4^% and a responsivity of 180 A W^−1^ under microwatt-level illumination. Furthermore, deposition of a thin MAPbBr_2_I layer on monolayer graphene enabled a photoconductive gain exceeding 10^9^ electrons per photon, leading to an extraordinary responsivity of 6.0 × 10^5^ A W^−1^ [58].

**Figure 6:**
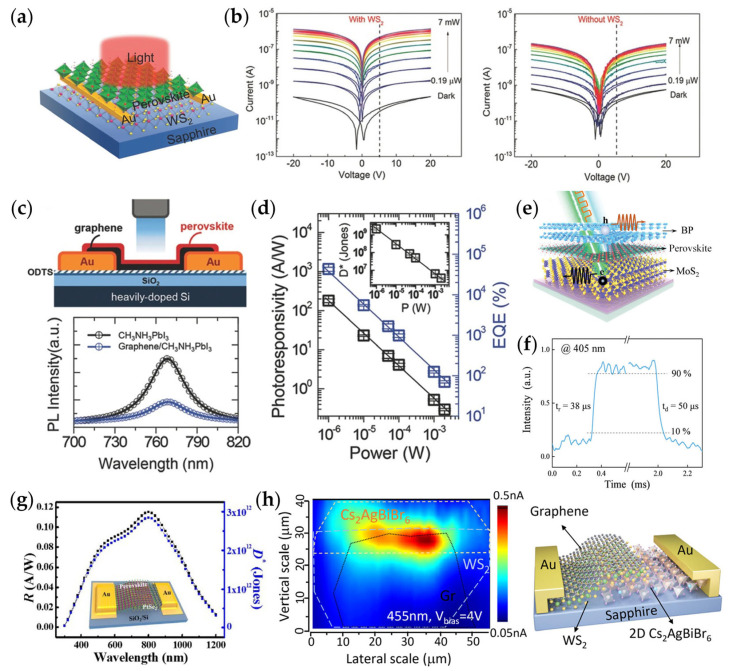
Structures and performance of MHP heterojunction photodetectors. (**a**) Schematic device structure of the hybrid WS_2_/perovskite photoconductor fabricated on a C-plane (0001) sapphire substrate; (**b**) I–V curves of the hybrid WS_2_/perovskite bilayer photoconductor measured in dark and under illumination with different white-light intensities [10]. (**c**) Schematic of a graphene/MAPbI_3_ hybrid photodetector, and PL spectra of the pristine MAPbI_3_ and graphene/MAPbI_3_ hybrid films upon excitation at 532 nm; (**d**) Variations in photoresponsivity and EQE with illumination power at 520 nm (V_GS_ = 0 V, V_DS_ = 0.1 V); inset: dependence of detectivity (D*) on illumination power [84]. (**e**) Diagram of the MoS_2_/(BA)_2_PbBr_4_/BP heterojunction photodetector, and (**f**) the time-resolved response signal of the device under 405 nm illumination, which are obtained from semiconductor analyzer and oscilloscope, respectively [86]. (**g**) Schematic of a multilayer PtSe_2_/Cs-doped FAPbI_3_ heterojunction device functioning as a broadband self-powered photodetector covering the ultraviolet to near-infrared range [87]. (**h**) Scanning photocurrent microscopy images (left) and schematic illustration (right) of the 2D Cs_2_AgBiBr_6_/WS_2_ photodetectors [88].

Several representative 2D MHP/TMDs heterojunction photoconductive devices are illustrated in Figure 6. In Figure 6a,b, the MAPbI_3_/WS_2_ heterojunction photodetector achieves an on/off ratio on the order of 10^5^ and a responsivity of approximately 17 A W^−1^, owing to the highly efficient interfacial charge transfer together with the mutually complementary electronic characteristics of MAPbI_3_ and WS_2_ [10], which is comparable to the responsivity of MoS_2_/metal-based photodetectors (16.65 A W^−1^) [85]. The response speed of this heterojunction is four orders of magnitude faster than that of pristine MAPbI_3_ films, underscoring the importance of interfacial engineering. As illustrated in Figure 6c,d, the graphene/MAPbI_3_ composite photodetector shows a remarkable improvement in UV–visible responsivity (up to 180 A·W^−1^) and EQE (~5 × 10^4^%), a result that stems from photoinduced electron injection from graphene into the perovskite, which effectively mitigates carrier recombination [84]. This mechanism was confirmed via PL quenching measurements. Building upon this concept, a CVD-grown double-layer photodetector using monolayer graphene as the substrate for the MAPbI_3_-PF absorber achieved D* ≈ 10^9^ Jones and broadband UV-visible detection capability.

Further advancements have been made using multi-component and vdWs heterojunctions. In Figure 6e,f, a BP/perovskite/MoS_2_ photodetector utilizes the photoreactive and dielectric properties of 2D MHP to achieve multimodal detection. Under 405 nm illumination, it exhibits a linear dynamic range of 100, rise/fall times of 38/50 µs, and a self-powered responsivity of 1.77 × 10^−2^ A W^−1^ [86]. At longer wavelengths, transient peak responses appear, enabling simultaneous multispectral detection and signal demultiplexing. Similarly, as shown in Figure 6g, a PtSe_2_/doped CsFAPbI_3_ heterojunction achieves broadband, self-driven photodetection, with a photo-to-dark current ratio of 5.7 × 10^3^, a responsivity of 1.177 × 10^2^ A W^−1^, and a zero-bias detectivity of ≈2.91 × 10^12^ Jones. The device also shows ultrafast rise/fall times (78/60 ns) and maintains stable performance for over three weeks in ambient conditions, indicating excellent air stability [87]. In addition, as illustrated in Figure 6h, a low-power Cs_2_AgBiBr_6_/WS_2_/graphene vertical heterojunction achieves a detectivity of ≈1.5 × 10^13^ Jones, rise/fall times of 52.3/53.6 µs, and an open-circuit voltage of ≈0.75 V, demonstrating the potential of lead-free perovskite heterojunctions in energy-efficient optoelectronic devices [88].

In summary, 2D MHP-based heterojunctions offer a versatile platform for next-generation photodetectors. Through rational interface design and material engineering, these hybrid systems achieve broadband, high-speed, and self-powered photodetection while effectively suppressing dark current and reducing power consumption. The synergistic combination of 2D MHPs and other vdW materials thus provides a promising route toward integrated, high-performance, and flexible optoelectronic technologies. Table 1 presents typical photoresponse results of MAPbX_3_-based heterojunction optoelectronic devices.

### 4.3. Light Emitting Diodes (LEDs)

MHPs exhibit excellent light emission characteristics in the visible to near-infrared regions, including high PL quantum yields, broad spectral tunability, and high color purity, making them important candidate materials for next-generation high-performance LEDs. However, the lower exciton binding energy in three-dimensional perovskites results in the easy dissociation of photogenerated excitons, limiting the efficiency of electron-photon conversion. To overcome this bottleneck, 2D MHPs with higher exciton binding energies have attracted attention. The quantum confinement effects in 2D MHPs significantly enhance the spatial confinement of carriers and radiative recombination, providing a new strategy for efficient light emission.

Some researchers reported the MHP-based LED using layered (PEA)_2_PbI_4_ perovskite, achieving brightness up to 10,000 cd m^−2^, showcasing the potential of low-dimensional MHPs in electroluminescence [95,96]. Nevertheless, the limited exciton binding energy in bulk perovskites continues to restrict device quantum efficiency. Strategies such as constructing ultra-thin active layers or utilizing low-dimensional perovskite emitters effectively confine carriers, thereby increasing the probability of radiative recombination and enhancing EQE.

**Figure 7:**
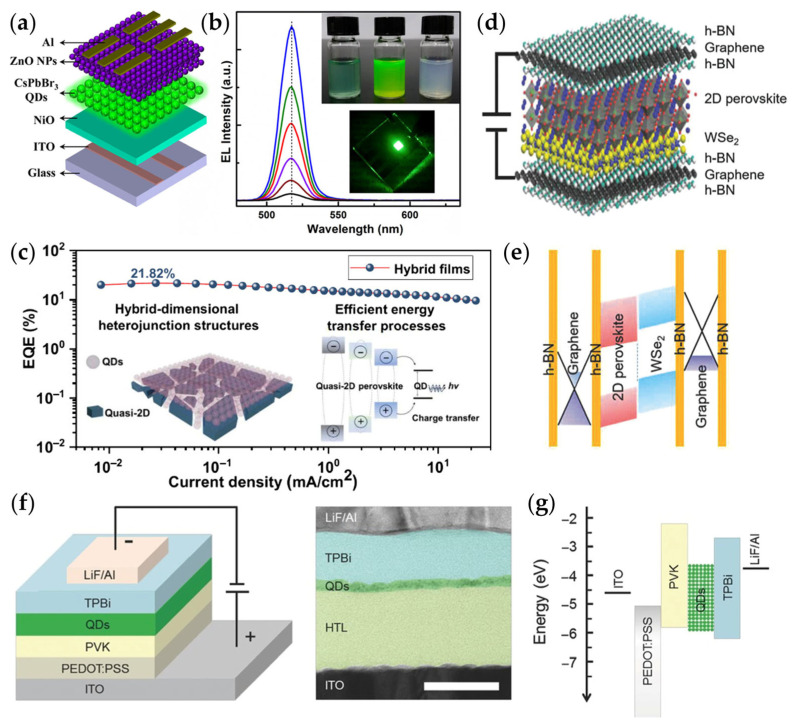
Structures and emission characteristics of perovskite LEDs with different architectures. (**a**) Schematic diagram of an all-inorganic CsPbBr_3_ QD heterojunction LED; (**b**) the electroluminescence spectra of the LED under different voltages. Inset (top): precursor solutions used for device fabrication, including C_10_H_14_NiO_4_ in acetonitrile, CsPbBr_3_ QDs in hexane, and ZnO nanoparticles in chlorobenzene; Inset (bottom): photograph of the operating LED (2 × 2 mm^2^ active area, driven at 5.0 V) [8]. (**c**) EQE versus current density of QD/quasi-2D perovskite hybrid films. Inset: Schematic of the positional relationship between QDs and quasi-2D perovskite and exciton transfer from quasi-2D perovskite into QDs with radiative recombination [97]. (**d**) Schematic drawing and (**e**) corresponding band diagram of the perovskite LED based on 2D heterojunction [98]. (**f**) The device structure and cross-sectional TEM image of multilayer perovskite-based LED device (Scale bar, 50 nm); (**g**) energy band alignment diagram of the perovskite-based LED in a p–i–n layered configuration [99].

Recent advances in perovskite LED research have focused on high-performance, low-cost solid-state lighting. In Figure 7a,b, Shi et al. [8] proposed a solution-processed all-inorganic heterojunction strategy, using n-ZnO nanoparticles and p-NiO as carrier injection layers to fabricate highly efficient CsPbBr_3_ QD LEDs with a peak brightness of 6093.2 cd m^−2^ and an EQE of 3.79%. The device maintained excellent stability under high humidity (75%, 12 h) and high temperature (393 K) conditions, significantly outperforming previously reported devices, offering an effective solution for achieving high-stability perovskite LEDs. Building on this, Li et al. [97] addressed the limitations of near-infrared (NIR, >750 nm) perovskite QD luminescence efficiency by stacking QDs with quasi-2D perovskite layers to construct a mixed-dimensional (0D/quasi-2D) structure (Figure 7c), with the quasi-2D phase acting as a protective and passivation layer. Femtosecond TA results showed that the mixed film exhibited a threefold longer lifetime compared to pure QD films, and the luminescence retained 90% of its initial intensity at 400 K. The NIR QD-LED based on this structure achieved an EQE of 22%, and the device lifetime was improved by approximately 6.5 times.

Inspired by the luminescence mechanisms of 2D material heterojunctions, researchers further proposed an atomic-level 2D perovskite quantum well LED design (Figure 7d,e) [98]. In these devices, precise stacking of RPP with h-BN and graphene layers enables band engineering and optical tuning. Controlling quantum well thickness enhances exciton confinement and radiative recombination, while transparent h-BN/graphene electrodes improve photon extraction. Additionally, the interlayer “self-packaging” effect significantly enhances device stability. As shown in Figure 7f,g, typical 2D perovskite LEDs use a sandwich-like p-i-n structure, with perovskite as the emission layer and carrier transport layers introduced to achieve balanced injection and enhanced light emission. By controlling the chemical composition and quantum well thickness, the emission spectrum can be tuned, and interface optimization further enhances electroluminescent efficiency [99]. Currently, the performance of 2D perovskite LEDs continues to improve: the EQE of polymer composite structures has reached 0.48%, energy funnel structures have increased to 8.8%, and self-organizing multiple quantum well structures have achieved breakthroughs up to 11.7% [100].

Moreover, combining quantum well stacking with tandem architectures enables highly efficient 2D perovskite tandem LEDs, potentially doubling monochromatic light emission efficiency or achieving white-light output through complementary emission layers. This innovative strategy provides a new direction for the application of MHPs in high-stability lighting and display fields, holding significant scientific and engineering importance.

### 4.4. Field-Effect Transistors (FETs)

**Figure 8:**
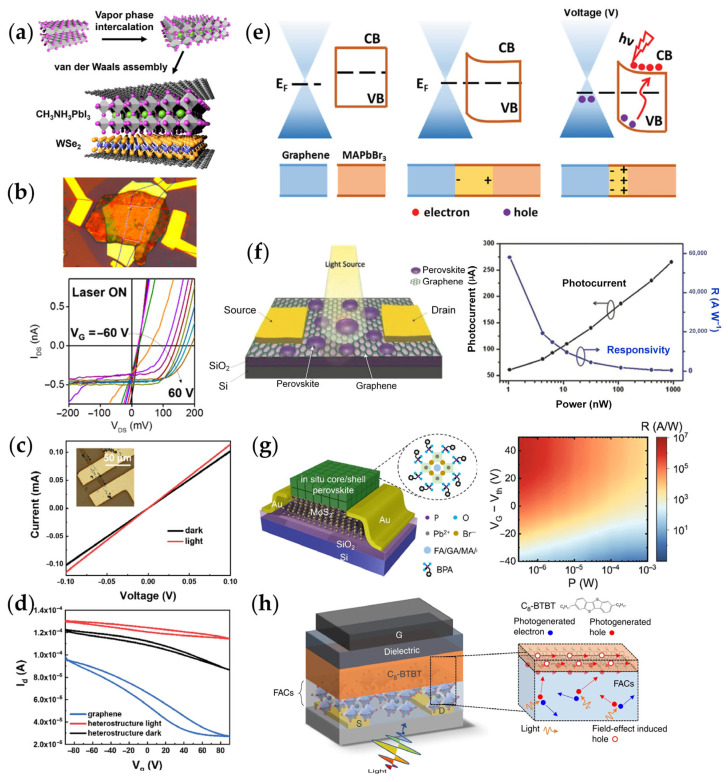
MHP-based heterojunction phototransistors. (**a**) Structure of the graphene/perovskite/WSe_2_/graphene multilayer heterojunction and (**b**) the corresponding FET transfer characteristics [101]. (**c**) I–V curves of MAPbBr_3_/graphene phototransistor under dark and white light conditions. Inset: device photo; (**d**) Transfer curves of graphene and MAPbBr_3_/graphene FETs measured at a source–drain voltage of 0.1 V under dark and light conditions; (**e**) Energy band diagrams of graphene and MAPbBr_3_ platelets before contact and under dark/light illumination, highlighting the depletion region at the interface [25]. (**f**) Schematic diagram of a MAPbBr_2_I/monolayer graphene heterojunction phototransistor, showing the variation in photocurrent and responsivity with illumination power at a wavelength of 405 nm [58]; (**g**) Schematic of PNCs/MoS_2_ phototransistor (left) and responsivity from shot-noise measurements under different gate voltages and laser powers at V_D_ = 0.1 V (right) [102]. (**h**) Illustration of the photocarrier transport mechanism in a type-I heterojunction phototransistor, with G, S, and D denoting the gate, source, and drain electrodes [103].

Figure 8 illustrates the application of several MHP heterojunctions in FETs devices and their performance characteristics. In Figure 8a,b, it is shown a schematic diagram of a graphene/perovskite/WSe_2_/graphene multilayer heterojunction and its corresponding FET characteristics [101]. This multilayer transistor exhibits not only a high switching ratio but also distinct gate-tunable diode characteristics and open-circuit voltage behavior. As the gate voltage is adjusted from −60 V to 60 V, the device transitions from a symmetric p-p junction to an n-p junction, demonstrating excellent point-control characteristics. This finding highlights the crucial role of carrier dynamics, particularly carrier transfer and modulation, in the heterojunction, driving the advancement of optoelectronic devices.

Figure 8c–e further explore the applications of vdW materials in optoelectronic devices [25]. By selectively growing MAPbBr_3_ flakes on monolayer graphene via chemical vapor deposition, a MHP/graphene heterojunction was fabricated. Raman spectroscopy indicated that the perovskite flakes p-type doped the graphene, lowering its Fermi level. Under illumination, the perovskite surface became negatively charged, further enhancing the p-type characteristics of graphene. These findings offer a better understanding of the strong interface coupling between perovskite and 2D materials and reveal the carrier transfer process at the heterojunction interface under illumination. These dynamic processes not only improve the photoconductive performance of the device but also provide new insights for the design of novel optoelectronic devices.

With further research into 2D MHP heterojunctions, Figure 8f emphasizes the influence of the morphology of perovskite thin films on the performance of optoelectronic devices. Wang et al. [58] reported a high-performance hybrid phototransistor, which consists of a monolayer of graphene covered with MAPbBr_2_I islands that have controllable island size and distribution. This significantly reduces the recombination rate of photogenerated carriers and improves the photoconductive gain (approximately 10^9^) and responsivity (approximately 6.0 × 10^5^ A W^−1^). The device’s performance benefits from the efficient photocarrier separation and transfer at the heterojunction interface, where holes are effectively transferred to the graphene layer while electrons are captured by the perovskite, creating a photogating effect. This phenomenon underscores the importance of dynamic processes, especially carrier separation and transfer at the interface, as key factors in achieving high optoelectronic performance.

To overcome the intrinsic limitation of low absorption in atomically TMDs, Figure 8g shows the potential application of TMDs in photodetectors [102]. While TMDs offer advantages in high integration and flexibility due to their thin-layer structure, their low light absorption efficiency limits the enhancement of optoelectronic performance. To address this issue, we propose a method to combine TMDs with perovskite nanocrystals (PNCs) to improve the surface defects of PNCs and enhance photocarrier injection, thereby optimizing the optoelectronic performance. The PNCs/MoS_2_ heterojunction adopts a type-II band alignment, effectively separating electrons and holes and generating significant photogating effects. The dynamic processes in this heterojunction, particularly the rapid separation and transfer of carriers at the interface, directly enhance the device’s photoconductive response (2.2 × 10^6^ A W^−1^) and specific detectivity (9.0 × 10^11^ Jones) [102]. This study not only demonstrates the potential of heterojunctions in photodetectors but also reveals how 2D materials can regulate carrier dynamics through heterojunctions, further enhancing the efficiency of optoelectronic devices.

Building on this, Liu et al.’s research [25] further validated the impact of interface interactions using a field-effect transistor based on a graphene and MAPbBr_3_/graphene heterojunction. Under illumination, the photoresponse of the heterojunction device was significantly enhanced, indicating effective hole transfer to the graphene, thereby improving the device’s photoconductive performance. This study further demonstrates that carrier transfer and interface coupling in heterojunctions play a crucial role in enhancing optoelectronic performance. Additionally, Figure 8h depicts the carrier transport mechanism in a type-I heterojunction phototransistor based on FACs/C8-BTBT [103]. Under weak gate fields, photogenerated holes accumulate and transfer efficiently at the interface without significant interlayer charge exchange, underscoring the function of type-I junctions in photocarrier generation and collection. Collectively, these studies demonstrate that tailored heterojunction design, coupled with controlled interfacial dynamics, plays a decisive role in advancing the performance of perovskite-based field-effect photonic devices.

In summary, MHP/graphene heterojunctions, with their unique interface physical properties and multidimensional tunability, hold great potential in future research of optoelectronic functional materials. The combination of the bandgap tunability of MHPs and the diverse characteristics of 2D materials not only expands the functional boundaries of optoelectronic devices but also provides a new pathway for the realization of high-performance, flexible, and tunable optoelectronic systems. However, the carrier transfer process and ultrafast dynamics in perovskite/graphene heterojunctions remain insufficiently understood. To elucidate the fundamental interfacial dynamics of charge separation and recombination, further studies combining femtosecond time-resolved spectroscopy would be highly desirable.

## 5. Summary and Outlook

We have discussed the recent advances in metal halide-based heterojunctions, with a focus on their ultrafast carrier dynamics and potential applications. Key scientific issues regarding the commercialization of MHP-based optoelectronic devices—such as exciton dynamics, non-radiative recombination losses, and the efficient working principles of optoelectronic conversion devices—still require further experimental validation and exploration. Employing novel optical techniques to gain a deeper understanding of the microscopic physical mechanisms of MHPs is essential. Currently, the poor stability and difficulty in preserving MHPs remain urgent challenges that need to be addressed. Combining MHPs with other 2D semiconductor materials not only holds promise for enhancing their structural and environmental stability but also boosts carrier separation and transport efficiency, significantly improving the overall performance of optoelectronic devices. This offers new research directions for the development of next-generation high-performance optoelectronic devices.

The emergence of MHP-based heterojunctions represents a transformative advancement in the design of phototransistors, providing a compelling solution to overcome the inherent limitations of single-material systems. By strategically combining complementary material properties, these heterojunctions achieve unprecedented improvements in optoelectronic detection performance, including enhanced sensitivity, reduced noise levels, faster response times, and improved operational stability. The synergistic effect between the ultra-high mobility channels and the strong absorption perovskite layers effectively addresses the longstanding trade-offs between detectivity, responsivity, dark current, noise level, response speed, and spectral range, which have hindered the development of advanced phototransistors.

## Figures and Tables

**Table 1:** Summary of key parameters of photodetectors based on MAPbX_3_ heterojunction.

Heterostructure Type	*R* [A W^−1^]	EQE/[%]	D* [Jones]	On/Off Ratio	Response Time [ms]	Test Conditions	Ref.
MAPbI_3_ film /graphene	180	≈5 × 10^4^%	≈10^9^	/	*t*_rise_: 87, *t*_decay_: 540	*λ*: 520 nm, *P*: 1 mW,	[84]
MAPbI_3_ film /graphene	≈2700	/	/	/	*t*_rise_: <50, *t*_decay_: <50	*λ*: 633 nm, *P*: 1 pW, *V*_bias_: 0.1 V	[89]
MAPbI_3_ microwire /graphene	2.2 × 10^−3^	/	1.78 × 10^5^	/	*t*_rise_: ≈68	*λ*: 375–785 nm, *P*: 13.5 mW cm^−2^, *V*_bias_: 0.01 V	[90]
MAPbI_3_ film/WS_2_	17	/	2 × 10^12^	3 × 10^5^	*t*_rise_: 2.7, *t*_decay_: 7.5	For *R*: *λ*: white light, *P*: 0.2 μW cm^−2^, *V*_bias_: 5 V For D*: *λ*: 505 nm, *P*: 0.5 mW cm^−2^, *V*_bias_: 5 V	[10]
MAPbI_3_ film/WSe_2_	110	2.5 × 10^4^%	2.2 × 10^11^	/	*t*_rise1_: 143, *t*_rise2_: 2113; *t*_decay1_: 225, *t*_decay2_: 2983	*λ*: 532 nm, *P*: 2.8 mW, *V*_bias_: 2 V	[91]
MAPbI_3_ film /2H-MoS_2_	142	3.5 × 10^4^%	/	≈300	*t*_rise_: <25, *t*_decay_: <50	*λ*: 500 nm, *P*: 31.3 μW cm^−2^, *V*_bias_: 2 V	[92]
MAPbI_3_ NC /MoS_2_	0.696	/	1.94 × 10^−12^	87.47	*t*_rise_: 50 (from 10 to 90%), *t*_decay_: 16 (from 90 to 10%)	*λ*: 532 nm, *P*: 51.5 μW cm^−2^, *V*_bias_: 3 V	[93]
MAPbBr_3_ NC /MoS_2_	5.6 × 10^−3^	/	/	1.41	*t*_rise_: 1650 (80%), *t*_decay_: 1200 (80%)	*λ*: 405 nm, *P*: 9.6 mW, *V*_bias_: 3 V	[94]

*V*_bias_: applied bias voltage; *V*_DS_: drain-source voltage; *V*_GS_: gate-source voltage.

## Data Availability

No new data were created or analyzed in this study. Data sharing is not applicable to this article.

## Abbreviations

The following abbreviations are used in this manuscript:

2D	Two-dimensional
MHPs	Metal halide perovskites
LEDs	Light-emitting devices
PSCs	perovskite solar cells
PLQY	Photoluminescence quantum yield
vdWs	van der Waals
h-BN	Hexagonal boron nitride
TMDs	Transition metal dichalcogenides
TA	Transient absorption
THz	Terahertz
PL	Photoluminescence
MA^+^	CH_3_NH_3_^+^
TEA^+^	(C_2_H_5_)_4_N^+^
CBM	Conduction band minimum
VBM	Valence band maximum
T_c_	Effective temperature
TRTS	Time-resolved THz spectroscopy
TRPL	Time-resolved photoluminescence spectroscopy
OPA	Optical parametric amplifier
PB	Photobleaching
SE	Stimulated emission
PIA	Photoinduced absorption
OPTP	Optical pump-THz probe
ETL	Electron transport layer
DFT	Density functional theory
HTL	Hole transport layer
RPP	Ruddlesden–Popper perovskites
QDs	Quantum dots
ITO	Indium tin oxide
PCE	Power conversion efficiency
EQE	External quantum efficiency
I–V	Current-voltage
D*	detectivity
NIR	Near-infrared
FETs	Field-Effect Transistors
NC	Nanocrystal
PNCs	Perovskite nanocrystals
